# Mother's and children's ADHD genetic risk, household chaos and children's ADHD symptoms: A gene–environment correlation study

**DOI:** 10.1111/jcpp.13659

**Published:** 2022-07-14

**Authors:** Jessica C. Agnew‐Blais, Jasmin Wertz, Louise Arseneault, Daniel W. Belsky, Andrea Danese, Jean‐Baptiste Pingault, Guilherme V. Polanczyk, Karen Sugden, Benjamin Williams, Terrie E. Moffitt

**Affiliations:** ^1^ Department of Psychology, School of Biological and Behavioural Sciences Queen Mary University London London UK; ^2^ Department of Psychology, School of Philosophy, Psychology and Language Sciences University of Edinburgh Edinburgh UK; ^3^ Social, Genetic, and Developmental Psychiatry Centre, Institute of Psychiatry Psychology, and Neuroscience, King's College London London UK; ^4^ Department of Epidemiology and Butler Columbia Aging Center Columbia University Mailman School of Public Health New York NY USA; ^5^ Promenta Center University of Oslo Oslo Norway; ^6^ Department of Child and Adolescent Psychiatry Institute of Psychiatry, Psychology and Neuroscience, King's College London London UK; ^7^ National and Specialist Child Traumatic Stress and Anxiety Clinic South London and Maudsley NHS Foundation Trust London UK; ^8^ Clinical, Educational and Health Psychology, Division of Psychology and Language Sciences University College London London UK; ^9^ Department of Psychiatry University of São Paulo Medical School São Paulo Brazil; ^10^ Department of Psychology and Neuroscience Duke University Durham NC USA

**Keywords:** ADHD, genetics, longitudinal studies, family factors, early life experience

## Abstract

**Background:**

Chaotic home environments may contribute to children's attention‐deficit hyperactivity disorder (ADHD) symptoms. However, ADHD genetic risk may also influence household chaos. This study investigated whether children in chaotic households had more ADHD symptoms, if mothers and children with higher ADHD genetic risk lived in more chaotic households, and the joint association of genetic risk and household chaos on the longitudinal course of ADHD symptoms across childhood.

**Methods:**

Participants were mothers and children from the Environmental Risk (E‐Risk) Longitudinal Twin Study, a UK population‐representative birth cohort of 2,232 twins. Children's ADHD symptoms were assessed at ages 5, 7, 10 and 12 years. Household chaos was rated by research workers at ages 7, 10 and 12, and by mother's and twin's self‐report at age 12. Genome‐wide ADHD polygenic risk scores (PRS) were calculated for mothers (*n* = 880) and twins (*n* = 1,999); of these, *n* = 871 mothers and *n* = 1,925 children had information on children's ADHD and household chaos.

**Results:**

Children in more chaotic households had higher ADHD symptoms. Mothers and children with higher ADHD PRS lived in more chaotic households. Children's ADHD PRS was associated with household chaos over and above mother's PRS, suggesting evocative gene–environment correlation. Children in more chaotic households had higher baseline ADHD symptoms and a slower rate of decline in symptoms. However, sensitivity analyses estimated that gene–environment correlation accounted for a large proportion of the association of household chaos on ADHD symptoms.

**Conclusions:**

Children's ADHD genetic risk was independently associated with higher levels of household chaos, emphasising the active role of children in shaping their home environment. Our findings suggest that household chaos partly reflects children's genetic risk for ADHD, calling into question whether household chaos directly influences children's core ADHD symptoms. Our findings highlight the importance of considering parent and child genetic risk in relation to apparent environmental exposures.

## Introduction

Children who grow up in chaotic households, characterised by lack of routine, excessive noise and instability, are more likely to experience adverse outcomes, including behaviour problems (Jaffee, Hanscombe, Haworth, Davis, & Plomin, 2012) and lower school achievement (Hanscombe, Haworth, Davis, Jaffee, & Plomin, 2011). Children may cope with noisy and overstimulating home environments by blocking out extraneous stimuli, which could translate to inattention in school settings (Jaffee et al., 2012). Additionally, children in unstable environments may learn to select an immediate reward over a less certain future reward, leading children to be less likely to delay gratification (Kidd, Palmeri, & Aslin, 2013). Thus, behaviours associated with ADHD may be reactions to, or exacerbated by, chaotic households. However, household chaos may also reflect parental or offspring genetic risk for ADHD.

**Figure 1:**
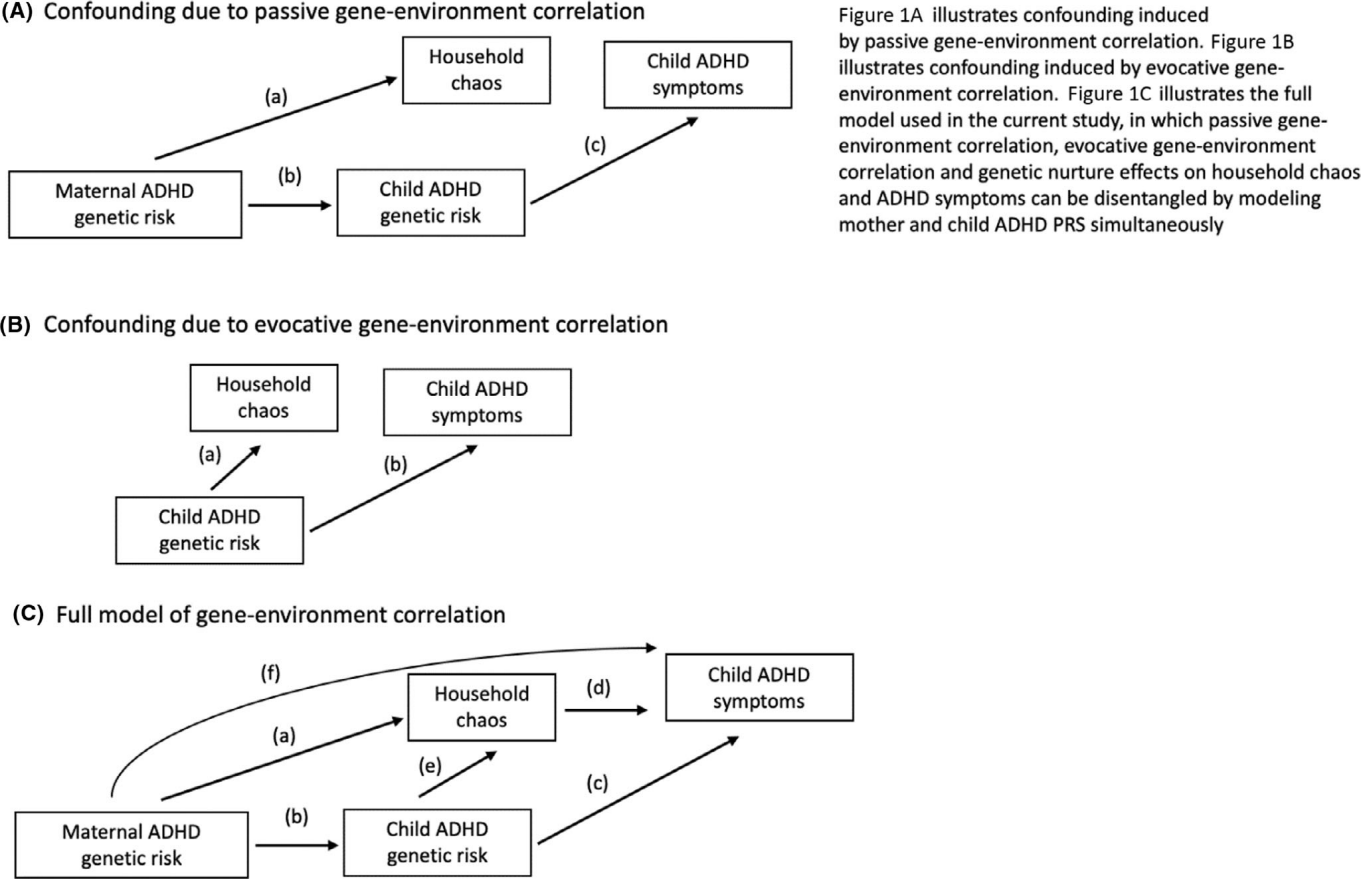
Gene–environment correlations of ADHD genetic risk with household chaos and child ADHD symptoms

Chaotic households are not randomly distributed in the population: parents at higher genetic risk for ADHD may create more household chaos, in addition to passing down more genetic variants associated with ADHD to their children. Likewise, children with higher ADHD genetic risk may increase levels of chaos at home. When genotype is associated both with children's outcomes, as well as environmental factors, this is an example of gene–environment correlation (Rutter, Moffitt, & Caspi, 2006). Gene–environment correlations can arise via several pathways, shown in the context of household chaos and ADHD in Figure 1. Figure 1A illustrates passive gene–environment correlation, in which maternal ADHD genetic risk is associated both with household chaos (pathway a) and child ADHD genetic risk (pathway b), which in turn is associated with ADHD symptoms (pathway c). Figure 1B illustrates evocative gene–environment correlation, in which child's ADHD genetic risk is associated both with household chaos (pathway a) and ADHD symptoms (pathway b). Gene–environment correlation can lead to confounding when genetic factors are common causes of both an environmental exposure and ADHD. Figure 1C illustrates how the current study can disentangle these types of gene–environment correlation, as well as an additional form of gene–environment interplay termed genetic nurture (Kong et al., 2018). Genetic nurture occurs if parents influence child outcomes via the environment they create, over and above the genes they pass on to their children. In the context of the current study, we can identify genetic nurture if mother's ADHD genetic risk influences children's ADHD symptoms after adjusting for the genes transmitted to children (i.e. children's ADHD genetic risk).

Until recently, estimation of gene–environment correlation was limited to studies characterised by a family design, for example twin studies. However, with the development of polygenic risk scores (PRS), these correlations can be examined in studies that include participant's genetic information. For example, recent research on gestational exposures found that mothers with higher ADHD PRS had more prenatal infections and acetaminophen use (Leppert et al., 2019), raising the possibility that some presumptively environmental prenatal exposures are influenced by genetics. Traditional twin studies are less well‐suited to examining gene–environment correlation associated with prenatal and household‐level exposures, as these are shared between twins. However, using genetic risk captured by PRS, we can examine gene–environment correlation outside of family‐based studies and for shared‐environmental exposures. In studies with both parental and offspring PRS, such as the current study, we can disentangle the contribution of gene–environment correlation conferred from parents (passive) and children (evocative).

In the current study, we investigate gene–environment correlation by examining household chaos and children's ADHD symptoms and testing several hypotheses. First, we examined whether children in more chaotic households showed higher ADHD symptoms across childhood. Second, we examined the extent to which household chaos was associated with ADHD genetic risk from mothers and children, and how mother's and children's genetic risk together may influence household chaos. Third, we investigated the joint association of mother's and children's ADHD genetic risk with household chaos on children's ADHD symptoms across childhood.

## Methods

### Study cohort

Participants were members of the Environmental Risk (E‐Risk) Longitudinal Twin Study, a birth cohort of 2,232 British children drawn from a larger birth register of twins born in England and Wales in 1994–95 (Trouton, Spinath, & Plomin, 2002). Briefly, the E‐Risk sample was constructed in 1999–2000 when 1,116 families with same‐sex 5‐year‐old twins participated in home‐visit assessments. This sample comprised 56% monozygotic (MZ) and 44% dizygotic (DZ) twin pairs; sex was evenly distributed within zygosity (49% male). Families were recruited to represent the UK population with newborns in the 1990s on the basis of residential location throughout England and Wales and mother's age.

Follow‐up home visits were conducted when children were aged 7 years (98% participation), 10 years (96%) and 12 years (96%). Home visits at ages 5–12 years included assessments with participants and their mother and were completed by trained research workers. Research workers had degrees in psychology, nursing or related disciplines, and received 1 month of intensive training as well as ongoing supervision by clinical researchers affiliated with the study. The Joint South London and Maudsley and the Institute of Psychiatry Research Ethics Committee approved each phase of the study. Parents gave written informed consent and twins gave assent between 5 and 12 years. At follow‐up, the study sample represents the full range of socioeconomic conditions in Great Britain, as reflected in the families' distribution on a neighbourhood‐level socioeconomic index (see Appendix S1. Methods Supplement).

### Household chaos

When the twins were aged 7, 10 and 12 years, research workers rated levels of household chaos based on observations during study visits as part of a questionnaire designed to gain an in‐depth understanding of the home environment and intrafamilial relationships of the participating families. This questionnaire capitalised on skills and knowledge of the research workers, who visited the home for approximately 2–4 hr. Research workers were trained to observe family interactions, personality traits of the parents and twins and the quality of the home environment. After the study visit, research workers answered questions adapted from the HOME measure (Bradley, Caldwell, Rock, Hamrick, & Harris, 1988) including: ‘Is the house chaotic or overly noisy?’ and ‘Does the child have a predictable daily schedule?’. Response options were ‘yes’/’a little’/’no’ (Table S1). Responses were summed. Participants missing more than half the items were coded as missing for the scale; scale scores of participants missing less than half the items were prorated. Internal consistency reliabilities ranged from α = .53 to α = .58 (Wertz et al., 2020).

When children were aged 12, mothers and twins were asked about household chaos with a scale assessing eight constructs: noise vs. quiet; hurry, rushing; misplaced belongings, messiness; unexpected people around, coming and going without notice; daily routine; predictability, dependability; conflict; and privacy. Twelve items adapted from the Confusion, Hubbub, and Order Scale (Matheny, Wachs, Ludwig, & Phillips, 1995), Family Routines Inventory (Jensen, James, Boyce, & Hartnett, 1983) and Family Ritual Questionnaire (Fiese & Kline, 1993) were mirrored for mothers and twins. Example items included: ‘The children[/I] have a set bedtime almost every night’ and ‘Even our big plans change at a moment's notice’. Response options were ‘not true’/’somewhat true’/‘very often or often true’ (Table S1). Responses were summed. Participants missing more than half the items were coded as missing for the scale; scale scores of participants missing less than half the items were prorated. Internal consistency reliabilities were α = .76 for mother's report and α = .78 for children's report (Wertz et al., 2020).

To create the combined age‐12 household chaos score, we standardised then averaged reports from research worker's impressions based on their observations during home visits with mothers, research worker's impressions based on their observations during home visits with twins, mother's self‐report and twin's self‐report. To create the total combined household chaos score, we averaged standardised chaos scales from ages 7, 10 and the combined age‐12 scale. Participants had to have information on household chaos from at least two ages to be included in the combined chaos score. The distribution of scores on the scales by age and rater is shown in Figure S1. Correlations between different raters at the same age ranged from *r* = .25 to *r* = .48 (Table S2). Correlations between household chaos ratings across age ranged from *r* = .48 to *r* = .56 (Table S3). The stability of household chaos across childhood was similar to that of other measures of household adversity, such as food insecurity (*r* = .51).

### Childhood ADHD symptoms

We ascertained childhood ADHD symptoms on the basis of mother‐ and teacher reports of 18 symptoms (9 symptoms of inattention and 9 symptoms of hyperactivity/impulsivity) derived from DSM‐IV diagnostic criteria (Kuntsi et al., 2004). Meeting diagnostic criteria required six or more symptoms of inattention or six or more symptoms of hyperactivity/impulsivity reported by mothers or teachers in the past six months, and the other informant must have endorsed at least two symptoms. In the current study, we examine mother‐reported ADHD symptoms in childhood.

### Genotyping and imputation

We used Illumina Omni Express 24 BeadChip arrays (twins: Version 1.1, mothers: Version 1.2; Illumina, Hayward, CA) to assay common single‐nucleotide polymorphism (SNP) variation in the genomes of cohort members and their mothers. We imputed additional SNPs using IMPUTE2 software (Howie, Donnelly, & Marchini, 2009). Additional details on imputation and genotyping are provided in Appendix S1. The E‐Risk cohort contains monozygotic twins, who are genetically identical; we therefore empirically measured genotypes of one randomly selected twin per pair and assigned these data to their monozygotic co‐twin. We directly measured genotypes of both members of dizygotic twin pairs. We restricted analyses to European‐descent participants (90% of the original E‐Risk cohort) because allele frequencies, linkage disequilibrium patterns and environmental moderators may vary across populations (Martin et al., 2017). Of the *n* = 1,116 E‐Risk families, there were *n* = 860 families for whom genetic data could be analysed, based on the mothers and at least one child having genetic data. There was no difference in the prevalence children's ADHD among families with and without genetic data (*p* = .54).

### Calculation of polygenic risk scores

Polygenic scoring was conducted following the method described by Dudbridge (2013) using PRSice (Euesden, Lewis, & O'Reilly, 2015). Briefly, SNPs reported in the most recent ADHD genome‐wide association study (GWAS) (Demontis et al., 2019) were matched with SNPs in E‐Risk. We used all available SNPs, irrespective of significance for their association with ADHD, to compute polygenic scores (no *p*‐value threshold was applied). We then performed clumping by retaining the SNP with the smallest *p*‐value from each LD block (excluding SNPs with *r* ^2^ > .1 in 500‐kb windows) and then weighted SNPs by effect estimate. To control for possible population stratification, we conducted a principal components analysis using PLINK v1.9 (Chang et al., 2015) and residualised polygenic scores for the first ten principal components. This PRS was normally distributed and standardised to mean zero and standard deviation of one.

### Statistical analysis

We first examined the association of total combined household chaos and children's ADHD symptoms at ages 7, 10 and 12 using linear regression, adjusting for child sex. To illustrate these associations, we categorised household chaos as quartiles and presented mean ADHD symptoms across these levels at ages 7, 10 and 12.

Next, we tested whether mother's ADHD PRS was associated with total combined household chaos, and with chaos reported by research workers when children were ages 7, 10 and 12, and by mothers and twins at age 12, using linear regression adjusting for child sex. We then further adjusted for child ADHD PRS to test for an effect of mother's ADHD PRS on household chaos over and above the genes they pass to their offspring. Next, we tested whether child's ADHD PRS was associated with total combined household chaos, and with chaos reported at different ages by different reporters, using linear regression adjusting for child sex. We then further adjusted for mother's ADHD PRS to test for evocative gene–environment correlation.

To examine the joint effect of mother's and children's ADHD PRS with total combined household chaos on children's ADHD symptoms, we used multilevel models to examine baseline level of, and change in, total, hyperactivity/impulsivity and inattention symptoms from ages 5 to 12. We examined ADHD symptom domains separately as we previously identified slightly different associations with ADHD PRS (Agnew‐Blais et al., 2021). Because the distribution of ADHD symptoms was highly skewed, we used negative binomial regression. Model 1 includes genetic risk (mother's and children's ADHD PRS), model 2 includes household chaos, and model 3 includes mother's and children's ADHD PRS and household chaos. All analyses adjusted for child sex and non‐independence of twin observations using the sandwich variance estimator in Stata (STATA, 2015).

In sensitivity analyses, we estimated the attenuation of the association of total combined household chaos and childhood ADHD symptoms adjusting for different strengths of gene–environment confounding using the GSens package in R (Pingault et al., 2020). A limitation of correcting for gene–environment confounding via PRS alone is that PRS explains only a small proportion of variance in ADHD symptoms (around 1–3% (Ronald, De Bode, & Polderman, 2021)). Thus, adjusting for observed PRS would not account for as much genetic effect as is thought to exist. We used structural equation modelling to estimate how increasingly predictive polygenic scores, for example based on SNP heritability, would affect the association between household chaos and ADHD symptoms. We selected SNP heritability estimates based on a GWAS meta‐analysis of ADHD symptoms assessed in cohort studies (SNP heritability = 8% (Middeldorp et al., 2016)) and from a GWAS meta‐analysis of ADHD case–control status (SNP heritability = 22% (Demontis et al., 2019)). Further details are provided in Appendix S1.

## Results

### Is household chaos associated with higher childhood ADHD symptoms?

**Figure 2:**
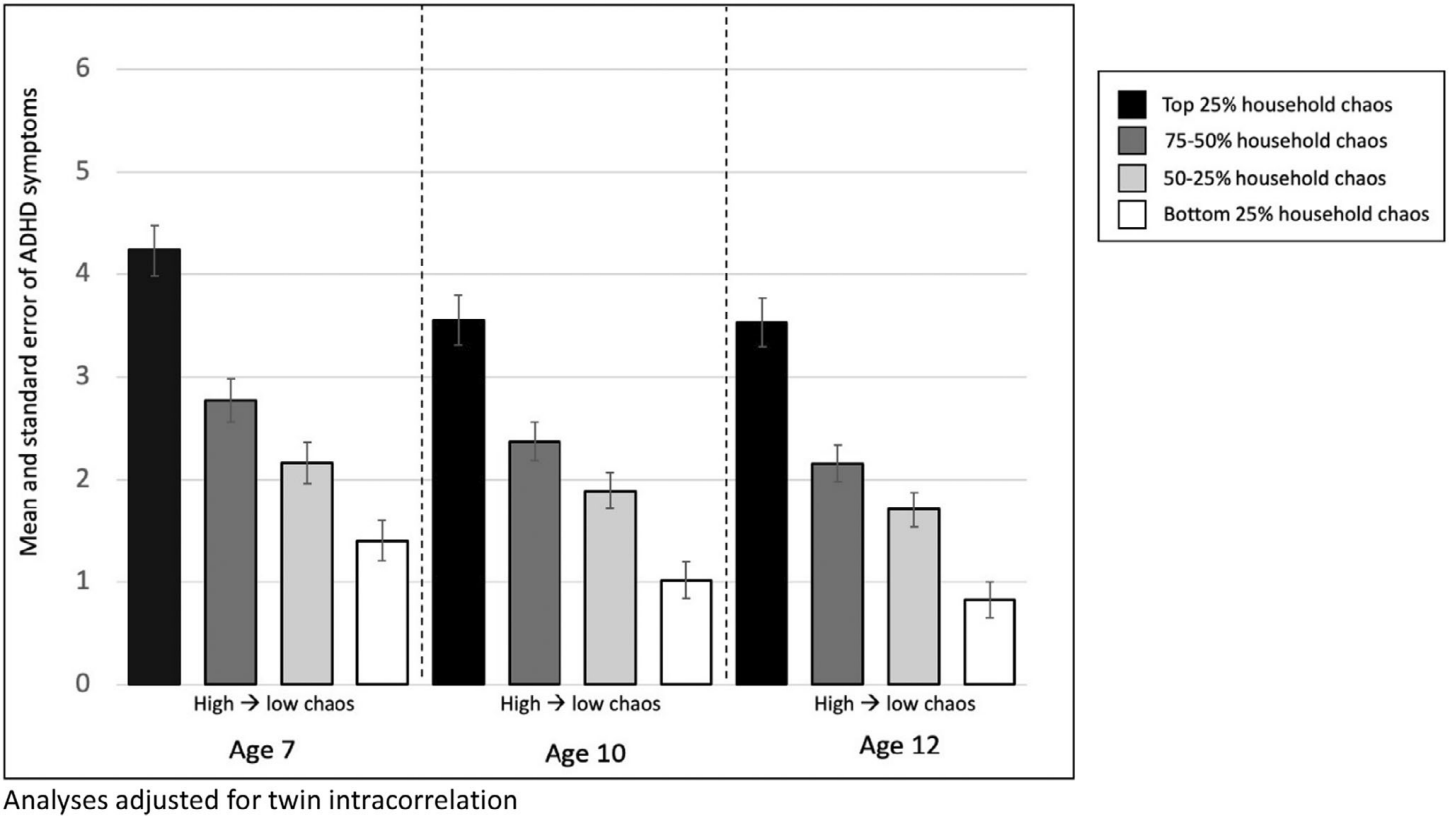
Mean ADHD symptoms at ages 7, 10 and 12 by quartile of household chaos

Higher levels of household chaos were associated with children's ADHD symptoms at ages 7, 10 and 12, with a standard deviation increase in household chaos associated with about one additional symptom (age 7 b = 1.13 *p* < .001, age 10 b = 0.95 *p* < .001, age 12 b = 1.09 *p* < .001). Figure 2 illustrates the dose–response relationship between household chaos and ADHD symptoms, with higher levels of household chaos associated with more ADHD symptoms across increasing chaos quartiles. Children in the highest household chaos quartile have nearly three additional ADHD symptoms compared with the lowest quartile.

### Do mothers and children with higher ADHD PRS live in more chaotic households?

**Figure 3:**
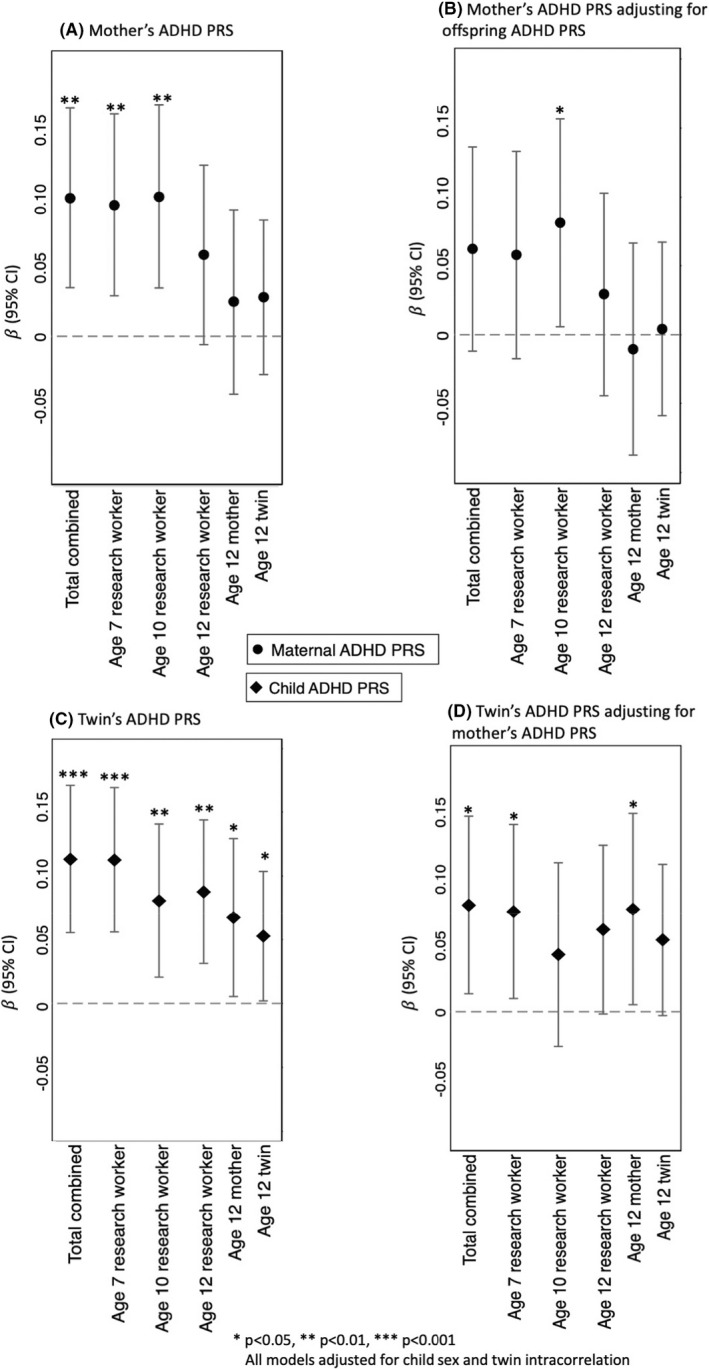
Standardised regression coefficients showing the association of mother's ADHD PRS (panels A and B) and twin's ADHD PRS (panel C and D) with household chaos at ages 7, 10 and 12 rated by study research workers, mothers and twins

We found evidence of gene–environment correlation, with mother's ADHD PRS significantly associated with total combined household chaos (β = 0.10, *p* = .003, Figure 3A). Looking separately by age and rater, we found that mothers with higher ADHD PRS were rated by research workers as having more chaotic households when children were ages 7 (β = 0.10, *p* = .005) and 10 (β = 0.10, *p* = .003). Mother's ADHD PRS was not associated with their own (β = 0.03, *p* = .463) or their children's (β = 0.03, *p* = .321) ratings of household chaos when children were age 12. Further adjusting for child's ADHD PRS (Figure 3B), associations were attenuated and mother's ADHD PRS was no longer associated with combined household chaos (β = 0.06, *p* = .100). This suggests that, overall, there is no effect of mother's ADHD PRS on household chaos over and above child's PRS. One exception is age 10, when mothers with higher ADHD PRS were rated by research workers as having more chaotic households, adjusting for child's PRS (β = 0.08, *p* = .035).

Children with higher ADHD PRS had higher total combined household chaos (β = 0.11, *p* < .001, Figure 3C). Looking by age and rater, children with higher ADHD PRS were rated by research workers as having more chaotic households at ages 7 (β = 0.11, *p* < .001), 10 (β = 0.08, *p* = .008) and 12 (β = 0.09, *p* = .002). When children were 12, mothers of children with higher ADHD PRS also rated their households as more chaotic (β = 0.07, *p* = .033) and children with higher ADHD PRS rated their own homes as more chaotic (β = 0.05, *p* = .042). Adjusting for mother's ADHD PRS (Figure 3D), the association between child's ADHD PRS and combined household chaos remained significant (β = 0.08, *p* = .019) providing evidence of evocative gene–environment correlation, that is children with higher ADHD genetic risk contribute to household chaos. Associations remained significant for research worker‐rated chaos at age 7, but not at ages 10 or 12. Mothers of children with higher ADHD PRS also rated their households as more chaotic when children were age 12, even accounting for mother's own PRS (β = 0.08, *p* = .035).

### Do ADHD PRS and household chaos have independent associations with the developmental course of ADHD symptoms?

Longitudinal models of ADHD symptoms examining the role of ADHD PRS found that children with higher PRS had more symptoms across childhood, even after accounting for mother's PRS (Table 1, Model 1: intercept IRR = 1.12, 95% CI 1.03, 1.22, *p* = .007). Mother's ADHD PRS was not associated with children's symptoms over and above the child's own PRS (intercept IRR = 1.07, 95% CI 0.99, 1.17, *p* = .11). This suggests a lack of genetic nurture; instead, mother's ADHD PRS is associated with children's ADHD symptoms via genes transmitted to their offspring rather than via the environments mothers create. While overall ADHD symptoms declined from age 5 to age 12, neither mother's nor child's ADHD PRS was associated with rate of change in ADHD symptoms (mother's slope IRR = 0.99, *p* = .25, child's slope IRR = 1.00, *p* = .52).

**Table 1:** Associations of mother's and child's ADHD PRS and household chaos with total ADHD, hyperactivity/impulsivity and inattention symptoms from age 5 to age 12

	Model 1, mother and child ADHD PRS		Model 2, household chaos		Model 3, PRS and household chaos	
	Intercept	Slope	Intercept	Slope	Intercept	Slope
*Total ADHD symptoms*	IRR	IRR	IRR	IRR	IRR	IRR
Mother's ADHD PRS	1.07	0.99			1.05	0.99
Child's ADHD PRS	1.12**	1.00			1.09*	1.00
Household chaos			1.47***	1.04***	1.43***	1.04***
*Hyperactivity/impulsivity*						
Mother's ADHD PRS	1.07	0.99			1.05	0.99
Child's ADHD PRS	1.13**	1.01			1.10*	1.01
Household chaos			1.40***	1.05***	1.35***	1.06***
*Inattention*						
Mother's ADHD PRS	1.06	0.99			1.02	0.99
Child's ADHD PRS	1.12	1.00			1.09	1.00
Household chaos			1.60***	1.02**	1.56***	1.02**

*p < .05, **p < .01, ***p < 0.001. IRR, incidence rate ratio; PRS, polygenic risk score. All analyses adjusted for twin intracorrelation and child's sex.

Children living in more chaotic households had higher ADHD symptoms (intercept IRR = 1.47, 95% CI 1.38, 1.57 *p* < .001), as well as slower rate of decline in symptoms (slope IRR = 1.04, 95% CI 1.03, 1.05, *p* < .001) (Table 1, model 2). Household chaos remained associated with ADHD symptom level and change after accounting for mother and child ADHD PRS (model 3, intercept IRR = 1.43, 95% CI 1.33, 1.53, *p* < .001, slope IRR = 1.04, 95% CI 1.03, 1.06, *p* < .001). Child's ADHD PRS also remained significantly associated with ADHD symptom level adjusting for household chaos (intercept IRR = 1.09, 95% CI 1.01, 1.18, *p* = .032). Results were similar examining hyperactivity/impulsivity and inattention symptoms separately; however, there was no association with ADHD PRS and inattention symptoms.

### Could the association between household chaos and ADHD symptoms be accounted for by genetic confounding?

**Figure 4:**
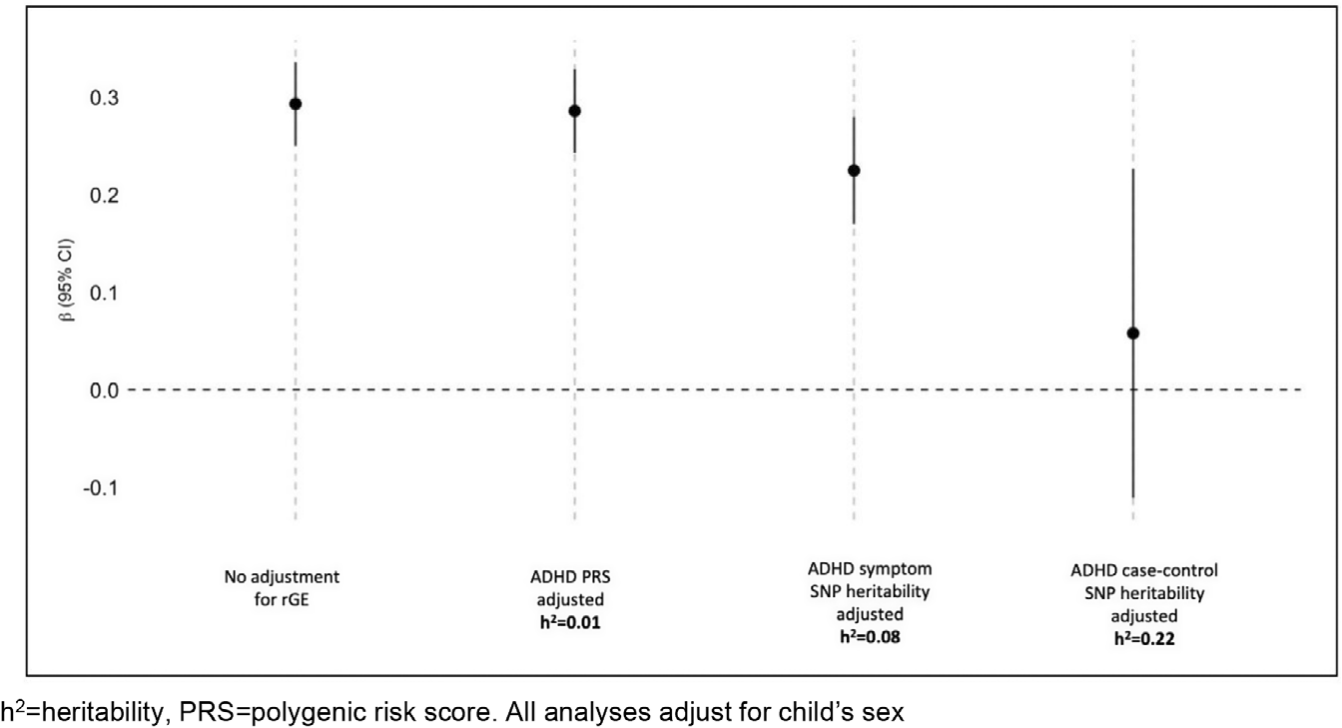
Estimating the effect of household chaos on ADHD symptoms adjusting for gene–environment confounding under different assumptions about heritability

We tested genetic confounding of associations between household chaos and childhood ADHD symptoms, accounting for different strengths of gene–environment confounding (Figure 4). We first adjusted only for child's PRS (accounting for about 1% of variance of ADHD symptoms). The association was slightly attenuated (β = 0.29, 95% CI 0.24, 0.33 *p* < .001), but remained significant, suggesting that household chaos is an environmental risk factor for childhood ADHD. However, because PRS are known to underestimate the full extent of genetic influences, we then adjusted for a magnitude of gene–environment confounding reflecting more realistic levels of genetic influence. When adjusting for the SNP heritability of general population ADHD symptoms (8% (Middeldorp et al., 2016)), the association between household chaos and ADHD was attenuated but remained significant (β = 0.23, 95% CI 0.17, 0.28 *p* < .001). Adjusting for SNP heritability estimated from case–control studies (22% (Demontis et al., 2019)), the association between household chaos and ADHD symptoms was reduced and no longer statistically significant (β = 0.06, 95% CI ‐0.11, 0.23, *p* = .50).

## Discussion

We found that children living in more chaotic households had higher ADHD symptoms across childhood. We also identified a significant contribution of child's ADHD genetic risk to levels of household chaos – over and above mother's ADHD genetic risk – suggesting evocative gene–environment correlation, such that children's genetic risk contributes to shaping their environments. Thus children with higher ADHD genetic risk are more likely to live in unpredictable, less organised, more chaotic households due at least in part to underlying genetic risk, most strongly from the genetic predisposition of the child. Studies examining environmental risk factors for ADHD that include participant genetic information should take into account the possibility of this kind of gene–environment correlation.

### Children's ADHD genetic risk contributes to household chaos

Our findings regarding an effect of children's ADHD PRS on household chaos emphasise the active role children play in shaping the family environment. Adoption and twin studies have identified such evocative gene–environment correlations, such as child effects on mother's anxiety (Ahmadzadeh et al., 2019). Recent work has extended this from family‐based studies to cohorts with genetic information and found that children's educational attainment PRS was associated with warmer, more sensitive and more cognitively stimulating parenting (Wertz et al., 2020). Here, we find that children's ADHD genetic risk is associated with chaos in the home reported by different raters at multiple ages across childhood, over and above mother's genetic risk.

That household chaos may be influenced by children's genes was identified in a previous twin study, which found children's perception of household chaos at ages 9 and 12 was heritable, estimated at 22% (Hanscombe, Haworth, Davis, Jaffee, & Plomin, 2010). In the current study, we find a role for child's genetics not just in their own perceptions of household chaos, but also in mother's perceptions and those of independent observers. Additionally, we identified that some of this genetic effect on household chaos was driven by ADHD genetic risk. Our findings regarding child effects on the family environment are consistent with research on the impact of ADHD medication on children's relationship with parents. Children with ADHD have been observed to have more negative interactions with their mothers. However, when children are effectively treated with ADHD medication, interactions with mothers improve (Schachar, Taylor, Wieselberg, Thorley, & Rutter, 1987). This suggests that some behaviours of children with ADHD may strain interactions with family members, emphasising the role of children in affecting family dynamics.

In the current study, we have information on mother's ADHD genetic risk only; thus, the association of children's ADHD genetic risk and household chaos over and above mother's genetic risk may also reflect father's genetic risk. Studies including genetic information on both parents can clarify whether this child effect may be due to residual genetic risk from fathers.

### No evidence of genetic nurture from mothers for children's ADHD symptoms

We did not find an effect of mother's ADHD PRS on children's ADHD symptoms over and above children's ADHD PRS. Thus, we do not find evidence for ‘genetic nurture’, whereby parents', in this case mothers', genetic predisposition influences children's outcomes via the environment parents create. Our finding is consistent with research using parent and child trios to create PRS from genetically transmitted and non‐transmitted alleles. A study using this approach found that only the genetically transmitted PRS was associated with offspring ADHD symptoms; thus, parental genetic predisposition affected children's ADHD symptoms via the genes they passed on, as opposed to the environment parents created (de Zeeuw et al., 2020). Our findings are also consistent with the near absence of shared‐environmental influences in twin analyses of ADHD symptoms, suggesting a limited role for shared home environment in ADHD incidence (Burt, 2009). Taken together, these findings suggest that genetic nurture may play a relatively limited role in childhood ADHD.

### Do chaotic households cause higher ADHD symptoms in children?

After accounting for child and mother ADHD PRS, higher household chaos was still associated with ADHD symptoms across development. However, adjusting for PRS alone likely does not fully adjust for the effect of genetic risk because PRS only captures about 1–3% of the variability in ADHD symptoms (Ronald et al., 2021), much less than other estimates of heritability which range from 8% to 80% (Middeldorp et al., 2016; Thapar, Harrington, Ross, & McGuffin, 2010). When we further adjusted the association of household chaos and ADHD symptoms to account for this additional genetic influence, household chaos was no longer significantly directly associated with children's symptoms. This suggests that household chaos may be more a reflection of children's ADHD genetic risk than a direct contributor to children's core ADHD symptoms.

These sensitivity analyses emphasise the need to consider the impact of gene–environment correlation on associations between apparent environmental risk factors and ADHD. As with all research, before drawing causal conclusions, it is important to triangulate findings by combining evidence from multiple study designs (Pingault et al., 2018). Our sensitivity analyses are dependent upon estimates of SNP heritability and are not, on their own, a sufficient test of whether household chaos is causally related to ADHD. Additionally, they are not as of yet adapted to longitudinal modelling, so do not address whether household chaos may affect change in ADHD over time. However, should future research confirm that household chaos does not play a causal role in ADHD, this could have clinical implications, including that interventions for ADHD should concentrate on improving children's symptoms themselves rather than focusing on the home environment.

### Objective measures of household chaos are more associated with ADHD genetic risk than mother and child‐report

We found that the link between ADHD genetic risk and household chaos was stronger with research worker report of household chaos than with report from mothers or children. This was surprising, as household chaos measures completed by mothers and children were more in‐depth than those completed by research workers. One explanation could be that individuals with a stronger genetic predisposition to ADHD are less sensitive to chaotic environments. Previous work has found that children's self‐report of chaotic environments is moderately heritable, suggesting that individual‐level genetic factors affect perceptions of chaos (Hanscombe et al., 2011). Children with ADHD tend to underreport their own impairment compared with external reporters (Owens, Goldfine, Evangelista, Hoza, & Kaiser, 2007), and it could be that those at higher risk for ADHD are similarly less aware of instability and unpredictability in their home environments. This points to the importance of considering source of information on household‐level risk factors.

### Strengths and limitations

There are several strengths of the current study, including longitudinal measurement of ADHD symptoms at four time points across childhood, allowing for examination of both level of and change in ADHD symptoms from childhood to early adolescence. Additionally, we can assess both passive and evocative gene–environment correlation because we have genetic information from mothers and children. Household chaos in our study is assessed by a range of informants, including trained researcher workers, thereby providing more objective assessments of household chaos and avoiding issues of shared method variance with the assessment of ADHD symptoms (reported by mothers). However, there are also several limitations. First, we focus on ADHD genetic risk in mothers and their offspring, but we lack genetic information on fathers. Thus, we cannot directly consider a potentially important part of the household environment or analyse transmitted and untransmitted alleles (Kong et al., 2018). Second, while the E‐Risk study has a twin design, household chaos is a largely shared exposure between siblings. Thus, we are unable to use additional twin analyses to disentangle household chaos and ADHD symptoms. Third, levels of household chaos are not perfectly stable across childhood: research worker‐rated chaos showed a slight mean decrease from age 7 (mean = 1.34, *SD* = 1.58) to age 10 (mean = 0.98, *SD* = 1.41) and to age 12 (mean = 0.97, *SD* = 1.37). While our goal was not to examine change in household chaos, supplemental analyses (Table S4, Figure S2) showing research worker‐rated household chaos from age 7 to 12 find that mother and child ADHD PRS were individually associated with level of household chaos, but only child ADHD PRS remained significantly associated when both PRS were included in the model. Neither mother's nor child's ADHD PRS was associated with change in household chaos.

## Conclusions

A wide range of environmental exposures have been associated with ADHD, from maternal smoking to children's TV viewing (Christakis, Zimmerman, DiGiuseppe, & McCarty, 2005; Langley, Rice, van den Bree, & Thapar, 2005). However, such associations must be considered in light of potential gene–environment correlation. Parents and children are not just passive recipients of environmental exposures, but actively shape their home environments. We found that children with a genetic predisposition to ADHD were more likely to live in homes characterised by noise, disorganisation and unpredictability. The opportunity to investigate this type of gene–environment correlation via molecular genetics is increasingly becoming available as cohort studies collect DNA from participants. Using polygenic risk scores and sensitivity analyses, here, we illustrate how observational studies with genetic information can investigate gene–environment correlation. Thus, gene–environment correlation can become not just a ubiquitous study limitation, but can be actively investigated and quantified to better understand environmental risk factors for ADHD and inform potential interventions.

## Supporting information


**Table S1** Chaos measure items by rater.
**Table S2.** Correlations between household chaos measures at each age by rater.
**Table S3.** Correlations between household chaos measures at each age.
**Table S4.** Association of mother's and child's ADHD PRS with household chaos as rated by study research workers from ages 7 to 12.
**Figure S1.** Distributions of scores on household chaos measures by age and rater, and for total combined household chaos.
**Figure S2**. Mean research worker rated household chaos from age 7 to 12 among those in the top quartile of child ADHD PRS and the bottom quartile of child ADHD PRS.
**Appendix S1.** Methods supplement.
