# Supplementary material for: Mother's and children's ADHD genetic risk, household chaos and children's ADHD symptoms: A gene–environment correlation study
Source: J Child Psychol Psychiatry. 2022 Jul 14;63(10):1153–63. doi: 10.1111/jcpp.13659 (PMC9796059; doi:10.1111/jcpp.13659)
Supplement: Supplementary file 1 — Table S1 Chaos measure items by rater. Table S2. Correlations between household chaos measures at each age by rater. Table S3. Correlations between household chaos measures at each age. Table S4. Association of mother's and child's ADHD PRS with household chaos as rated by study research workers from ages 7 to 12. Figure S1. Distributions of scores on household chaos measures by age and rater, and for total combined household chaos. Figure S2. Mean research worker rated household chaos from age 7 to 12 among those in the top quartile of child ADHD PRS and the bottom quartile of child ADHD PRS. Appendix S1. Methods supplement. [file JCPP-63-1153-s001.docx]

**Table S1.** Chaos measure items by rater

| Research worker observation | | | Mother-report | | | Twin-report | | |
| --- | --- | --- | --- | --- | --- | --- | --- | --- |
| Age | Items | Responses | Age | Items | Responses | Age | Items | Responses |
| 7, 10, 12 | (1) Is the house chaotic or overly noisy?  (2) Is the use of the TV appropriate?  (3) Do children have a predictable daily schedule? | 0 No  1 A little/ somewhat  2 Yes | 12 | (1) The atmosphere in our home is restful and calm  (2) We have a quiet time for the children to do homework each day  (3) The children have a set bed time almost every night  (4) If something is going to happen, we explain it to the children in advance  (5) We are always losing things at home  (6) We always seem to be rushed, in a hurry  (7) We always seem to be late  (8) Even our big plans change at a moment's notice  (9) You can hardly hear yourself think in our home  (10) Family members have very little privacy  (11) People outside the household constantly come and go here  (12) When you come home, you never know who will be here | 0 Not true  1 Somewhat or sometimes true  2 Very true or often true | 12 | (1) Our home is a restful and calm place  (2) I have a quiet time to do my homework each day  (3) I go to bed at the same time almost every school night  (4) If something is going to happen my parents tell me beforehand  (5) We are always losing things at home  (6) We always seem to be rushed and in a hurry  (7) We always seem to be late for things  (8) Even our big plans change without much warning  (9) You can hardly hear yourself think in our home  (10) I don’t have much privacy in my home  (11) People who don’t live here are always coming and going  (12) When I come home, I never know who will be here | 0 Not true  1 Somewhat or sometimes true  2 Very true or often true |

| Age and rater | Age 7 research worker observation | Age 10 research worker observation | Age 12 research worker observation, mother home visit | Age 12 research worker observation, twin home visit | Age 12 mother | Age 12 twin |
| --- | --- | --- | --- | --- | --- | --- |
| Age 7 research worker observation | 1.0 |  |  |  |  |  |
| Age 10 research worker observation | 0.481 | 1.0 |  |  |  |  |
| Age 12 research worker observation, mother home visit | 0.471 | 0.522 | 1.0 |  |  |  |
| Age 12 research observation, twin home visit | 0.446 | 0.529 | 0.728 | 1.0 |  |  |
| Age 12 mother | 0.348 | 0.341 | 0.480 | 0.375 | 1.0 |  |
| Age 12 twin | 0.229 | 0.226 | 0.268 | 0.253 | 0.307 | 1.0 |

**Table S2.** Correlations between household chaos measures at each age by rater

**Table S3.** Correlations between household chaos measures at each age

|  | Age 7 (research worker rated) | Age 10 (research worker rated) | Age 12 (combined research worker, mother, twins) |
| --- | --- | --- | --- |
| Age 7 (research worker rated) | 1.0 |  |  |
| Age 10 (research worker rated) | 0.478 | 1.0 |  |
| Age 12 (combined research worker, mother, twins) | 0.490 | 0.561 | 1.0 |

**Figure S1.** Distributions of scores on household chaos measures by age and rater, and for total combined household chaos

**
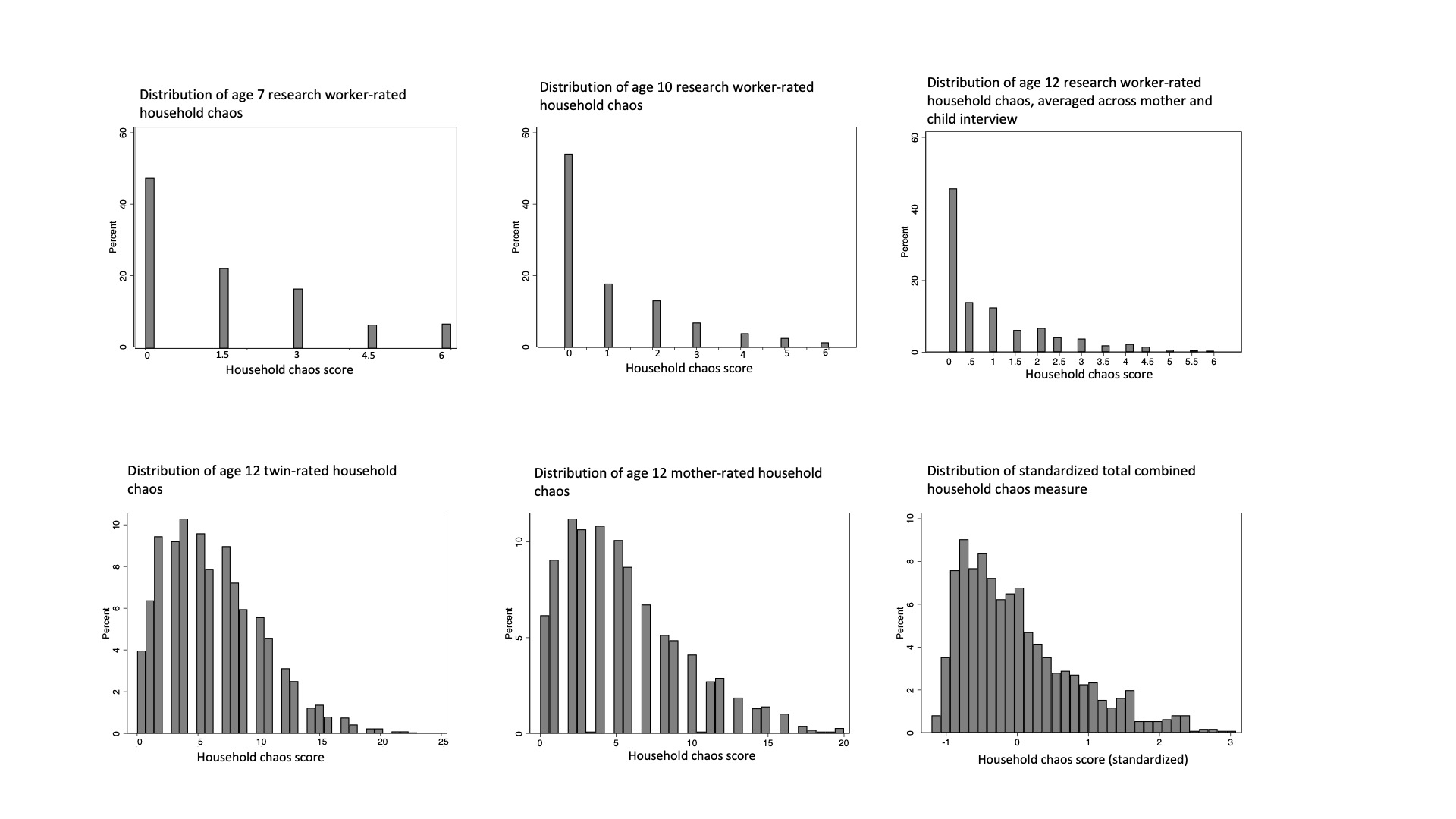
**

**Table S4.** Association of mother’s and child’s ADHD PRS with household chaos as rated by study research workers from ages 7 to 12

|  | Model 1, mother ADHD PRS | | Model 2, child ADHD PRS | | Model 3, mother and child ADHD PRS | |
| --- | --- | --- | --- | --- | --- | --- |
|  | Intercept | Slope | Intercept | Slope | Intercept | Slope |
|  | IRR | IRR | IRR | IRR | IRR | IRR |
| Mother’s ADHD PRS | 1.13** | 1.00 |  |  | 1.08 | 1.00 |
| Child’s ADHD PRS |  |  | 1.15*** | 1.00 | 1.10* | 1.00 |

* p<0.05, ** p<0.01, *** p<0.001. IRR=incidence rate ratio, PRS=polygenic risk score

All analyses adjusted for twin intracorrelation and child’s sex

**Supplemental Figure S2.** Mean research worker rated household chaos from age 7 to 12 among those in the top quartile of child ADHD PRS and the bottom quartile of child ADHD PRS

**
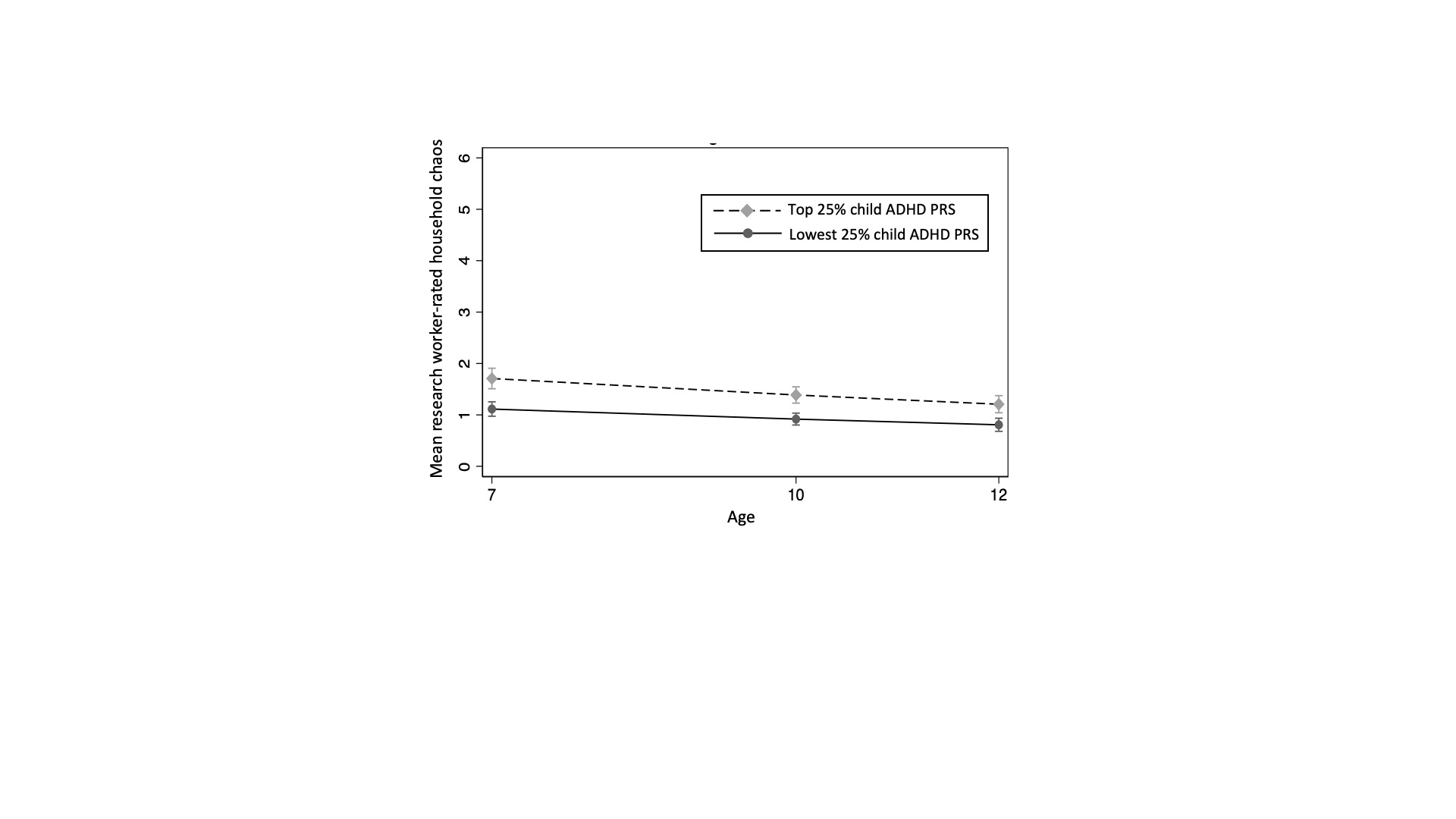
**

**Supplement 2. Methods supplement**

*Study cohort*

Participants were members of the Environmental Risk (E-Risk) Longitudinal Twin Study, a birth cohort of 2,232 British children drawn from a larger birth register of twins born in England and Wales in 1994-95 (Trouton, Spinath, & Plomin, 2002). Briefly, the E-Risk sample was constructed in 1999-2000 when 1,116 families with same-sex 5-year-old twins participated in home-visit assessments. This sample comprised 56% monozygotic (MZ) and 44% dizygotic (DZ) twin pairs; sex was evenly distributed within zygosity (49% male). Families were recruited to represent the UK population with newborns in the 1990s on the basis of residential location throughout England and Wales and mother’s age.

Follow-up home visits were conducted when children were aged 7 years (98% participation), 10 years (96%), and 12 years (96%). Home visits at ages 5-12 years included assessments with participants and their mother and were completed by trained research workers. Research workers had degrees in psychology, nursing, or related disciplines, and received one month of intensive training as well as on-going supervision by clinical researchers affiliated with the study. The Joint South London and Maudsley and the Institute of Psychiatry Research Ethics Committee approved each phase of the study. Parents gave written informed consent and twins gave assent between 5-12 years. The study sample represents the full range of socioeconomic conditions in Great Britain, as reflected in the families’ distribution on a neighborhood-level socioeconomic index (A Classification of Residential Neighborhoods, developed by CACI, Inc.; (Odgers, Caspi, Bates, Sampson, & Moffit, 2012; Odgers, Caspi, Russell, Sampson, Arseneault et al., 2012) 25.6% of E-Risk families live in “wealthy achiever” neighborhoods, compared with 25.3% nationwide; 5.3% compared with 11.6% in “urban prosperity” neighborhoods; 29.6% compared with 26.9% in “comfortably off” neighborhoods; 13.4% compared with 13.9% in “moderate means” neighborhoods; and 26.1% compared with 20.7% in “hard-pressed” neighborhoods. “Urban prosperity” families are underrepresented because such households are often childless.

*Genotyping and imputation*

We used Illumina Omni Express 24 BeadChip arrays (twins: Version 1.1, mothers: Version 1.2; Illumina, Hayward, CA) to assay common single-nucleotide polymorphism (SNP) variation in the genomes of cohort members and their mothers. We imputed additional SNPs using the IMPUTE2 software (Version 2.3.1; <https://mathgen.stats.ox.ac.uk/impute/impute_v2.html> (Howie, Donnelly, & Marchini, 2009)) and the 1000 Genomes Phase 3 reference panel (1000 Genomes Project Consortium, Abecasis, Auton, Brooks, DePristo et al., 2012). Imputation was conducted on autosomal SNPs appearing in dbSNP (Version 140; http://www.ncbi.nlm.nih.gov/SNP/(Sherry, Ward, Kholodov, Baker, Phan et al., 2001)) that were “called” in more than 98% of the samples. Invariant SNPs and SNPs with low minor allele frequency (<1%) were excluded. The E-Risk cohort contains monozygotic twins, who are genetically identical; we therefore empirically measured genotypes of one randomly-selected twin per pair and assigned these data to their monozygotic co-twin. MZ status was confirmed using genotypic data and SNPs from DNA methylation data for subsets of the sample. We directly measured genotypes of both members of dizygotic twin pairs. Prephasing and imputation were conducted using a 50-million-base-pair sliding window. We restricted our analyses to European-descent study participants (90% of E-Risk participants) because allele frequencies, linkage disequilibrium patterns, and environmental moderators of associations may vary across populations (Martin, Gignoux, Walters, Wojcik, Neale et al., 2017). Of the N = 1,116 E-Risk families, there were n = 860 families for whom genetic data could be analysed, based on the mothers and at least one child having genetic data. There were no differences in prevalence of meeting ADHD criteria among families with and without genetic data (p=0.54).

**Sensitivity Analysis using GSens**

In order to estimate the strength of rGE reflecting ADHD SNP heritability, we used the GSENS package (<https://github.com/JBPG/Gsens>, (Pingault, Rijsdijk, Schoeler, Choi, Selzam S et al., 2020). Within the GSENS package, we have specifically used the "gsensY" function, which is used when the analyses are based on one polygenic score for the outcome (Y) rather than multiple scores.

First, we tested the association between household chaos (X) and child ADHD symptoms (Y) after controlling for the polygenic score for ADHD symptoms.

This required specifying 5 parameters:

**rxy** = the observed phenotypic correlation between exposure X (household chaos) and outcome Y (children’s ADHD symptoms at age 5, age 7, age 10 and age 12); averaging across ages 5, 7, 10 and 12 this was r= 0.2933.

**rgx** = the observed correlation between phenotype X (household chaos) and the observed polygenic score for ADHD; this was r= 0.1129.

**rgy** = the observed correlation between phenotype Y (children’s ADHD symptoms at age 5, age 7, age 10 and age 12) and the observed polygenic score for ADHD (adjusted for sex and the first ten principal components); averaging across ages 5, 7, 10 and 12 this was r= 0.0985.

**n** = sample size (n=1953; this is the n of those with information on child ADHD PRS and ADHD symptoms)

**h2** = the variance explained in the outcome, here by the observed polygenic score (hence why h2 is **rgy^2**); this is h2=0.009 (about 1% of the variance in ADHD symptoms).

The results give three outputs including (1) adjusted Bxy, which is the standardized estimate of the relationship between household chaos and ADHD symptoms, adjusted for PRS (i.e. the residual association between household chaos and ADHD symptoms adjusting for the offspring's polygenic score); (2) an estimate of genetic confounding and (3) the total effect, which is the observed initial association between X and Y with no constraints.

Our output for this part of the model is listed below:

est se z pvalue ci.lower ci.upper

Adjusted Bxy 0.286 0.022 13.031 8.1143e-39 0.243 0.329

Genetic confounding 0.007 0.001 7.131 9.9888e-13 0.005 0.009

Total effect 0.293 0.022 13.553 7.651e-42 0.251 0.336

We then implemented the sensitivity analyses to examine genetic confounding under scenarios in which polygenic scores explain SNP-heritability in ADHD symptoms. We did this by adding to the "h2" option the chosen heritability estimate. We applied two estimates of SNP heritability, from the most recent GWAS of ADHD in cohort studies (SNP heritability=8% (Middeldorp, Hammerschlag, Ouwens, Groen-Blokhuis, St. Pourcain et al., 2016) and from the most recent published GWAS of ADHD case control status (SNP heritability=22% (Demontis, Walters, Martin, Mattheisen, & Alsa, 2019)).

The same parameters as above were specified for rxy, rgx, rgy and n, but variance explained by the observed polygenic score (the h2 parameter) was replaced with 0.08 in the first instance and 0.22 in the second instance:

Results for Middeldorp SNP heritability (SNP heritability=8%) model:

est se z pvalue ci.lower ci.upper

Adjusted Bxy 0.225 0.028 8.039 9.0468e-16 0.170 0.280

Genetic confounding 0.068 0.013 5.187 2.1331e-07 0.042 0.094

Total effect 0.293 0.022 13.485 1.9119e-41 0.251 0.336

The results show that under a SNP heritability scenario of h^2^=8%, the effect of household chaos on ADHD symptoms is attenuated (B=0.225) relative to when controlling for observed polygenic scores (B=0.286). These results are shown in Figure 4 in the main body of the paper.

Results for Demontis SNP heritability (SNP heritability=22%) model:

est se z pvalue ci.lower ci.upper

Adjusted Bxy 0.058 0.086 0.670 0.50256 -0.111 0.227

Genetic confounding 0.235 0.080 2.949 0.003192 0.079 0.392

Total effect 0.293 0.023 12.799 1.6569e-37 0.248 0.338

The results show that under a SNP heritability scenario of h^2^=22%, the effect of household chaos on ADHD symptoms is attenuated (B=0.06) and no longer reaches statistical significance (p=0.50). These results are shown in Figure 4 in the main body of the paper.

References

1000 Genomes Project Consortium, Abecasis, G.R., Auton, A., Brooks, L.D., DePristo, M.A., Durbin, R.M., Handsaker, R.E., Kang, H.M., Marth, G.T., & McVean, G.A. (2012). An integrated map of genetic variation from 1,092 human genomes. *Nature, 491*(7422), 56-65.

Demontis, D., Walters, R.K., Martin, J., Mattheisen, M., & Alsa, T.D. et al (2019). Discovery of the first genome-wide significant risk loci for ADHD. *Nature Genetics, 51*(1), 63-75.

Howie, B.N., Donnelly, P., & Marchini, J. (2009). A flexible and accurate genotype imputation method for the next generation of genome-wide association studies. *PLoS Genetics, 5*(6), e1000529.

Martin, A.R., Gignoux, C.R., Walters, R.K., Wojcik, G.L., Neale, B.M., Gravel, S., & Kenny, E.E. (2017). Human demographic history impacts genetic risk prediction across diverse populations. *American Journal of Human Genetics, 100*, 635–649.

Middeldorp, C., Hammerschlag, A., Ouwens, K., Groen-Blokhuis, M., St. Pourcain, B., & Greve, C., et al. (2016). A genome-wide association meta-analysis of attention-deficit/hyperactivity disorder symptoms in population-based pediatric cohorts. *Journal of the American Academy of Child and Adolescent Psychiatry, 55*(10), 896-905.

Odgers, C.L., Caspi, A., Bates, C.J., Sampson, R.J., & Moffit, T.E. (2012). Systematic social observation of children’s neighborhoods using Google Street View: a reliable and cost-effective method. *Journal of Child Psychology and Psychiatry, 53*(10), 1009-1017.

Odgers, C.L., Caspi, A., Russell, M.A., Sampson, R.J., Arseneault, L., & Moffit, T.E. (2012). Supportive parenting mediates neighborhood socioeconomic disparities in children's antisocial behavior from ages 5 to 12. *Developmental Psychopathology, 24*(3), 705-721.

Pingault, J., Rijsdijk, F., Schoeler, T., Choi, S., Selzam S, Krapohl, E., O'Reilly, P., & Dudbridge, F. (2020). Genetic sensitivity analysis: adjusting for genetic confounding in epidemiological associations. *PLOS Genetics, 17*(6), e1009590.

Sherry, S.T., Ward, M.H., Kholodov, M., Baker, J., Phan, L., Smigielski, E.M., & Sirotkin, K. (2001). dbSNP: the NCBI database of genetic variation. *Nucleic Acids Research, 29*(1), 308-311.

Trouton, A., Spinath, F., & Plomin, R. (2002). Twins Early Development Study (TEDS): a multivariate, longitudinal genetic investigation of language, cognition and behavior problems in childhood. *Twin Research, 5*(5), 444-448.
